# 

**Published:** 1917-04-07

**Authors:** 


### CONTENTS.

| | PAGE |
|---|---|
| **Easter Day 1917: The Murder of Non-Combatants** ... | 1 |
| **Municipalities and the Voluntary Principle** ... | 2 |
| **Hospital and Institutional News** ... | 3 |
| The Postmaster-General and the Saturday Fund—University College Hospital Meeting—Some Generous Benefactions of the Week The Pay of Superintendents of Civil War Hospitals—The Annual Report—Significant Facts—Large Type—The Office and Salary of Hospital Chaplains—The Last Lap at Leeds—A Remarkable Gift to a Hospital—Insanity and the War—Bread upon the Waters—Women's Hospital Unit in Russia—Looking Ahead to a Pension—A Record of Soldiers' Songs—A Recurring Difficulty for Hospital Secretaries—No Discretion for Nurses—This Week's Drug Market. | |
| **Disseminated Sclerosis** ... | 7 |
| A Knotty Problem of Causation and Treatment. | |
| **The Operative Treatment of Inguinal Hernia in Hospital** ... | 8 |
| **The Institutional Library** ... | 9 |
| The Effect of Printing on Literature. | |
| An Official Document to be Studied. | |
| **Leicester Royal Infirmary** ... | 12 |
| A Largely Attended Annual Meeting. | |
| **Parliament and the Profession** ... | 13 |
| **Hospital and Institutional Results** ... | 13 |
| **The Editor's Letter-Box** ... | 14 |
| Manipulative Surgery. | |
| **The Matrons' and Sisters' Department** ... | 15 |
| The Profession of British Nurses: II. Plain Words on Registration. | |
| Round the Hospitals. | |
| The Poor-Law Manner—Lord Rhondda's Opportunity—A Sister's Heroism at a Fire—Boxing: Nurses *v.* Patients?—British Nurse Attacked by Patient—A Way Out for the Partially Trained. | |
| **Some Problems of To-day** ... | 17 |
| II. Two Sorts of Waste of Children. | |
| **Gifts and Bequests** ... | 18 |
| **Matrons' and Sisters' Appointments** ... | vi |
| **Passing Events and Topics...** ... | vi |
| **The Book Market** ... | vi |
| **The Institutional Worker** ... (Suppl.) | 1 |
| Prevention of Waste in Foodstuffs. | |
| Question for April. | |
| Enquiries and Answers. | |
| The Sick and in Need. | |
| Employment and Training. | |
| Miscellaneous. | |

